# The ocular anterior segment examination of perinatal newborns by wide-field digital imaging system: a cross-sectional study

**DOI:** 10.1186/s12886-023-03139-1

**Published:** 2023-10-12

**Authors:** Yu-jing Wang, Min Ke, Ming Yan

**Affiliations:** Dept of ophthalmology, Zhongnan Hospital of Wuhan University, 430060 Wuhan, Hubei Province P. R. China

**Keywords:** Anterior segment, Neonates, Imaging system, Iris, Anterior chamber angle

## Abstract

**Purpose:**

The aim of this study was to evaluate and summarize the developmental rules of the ocular anterior segment of neonates by means of wild-field digital imaging system.

**Methods:**

We used the wide-field digital imaging system to sequentially capture images of the neonates’ eyes within 42 days after delivery, including the ocular surface, anterior segment, and fundus. At the same time, basic information at the time of birth and examination was collected.

**Results:**

Among 248 newborns, 51.21% were male. Abnormalities of the anterior segment such as visualization of anterior chamber angle vessels (79.03%) and iris vessels (51.21%), iris process (42.34%), persistent pupillary membranes (19.35%), albinism, congenital cataracts, corneal leucoma, and subconjunctival hemorrhage were observed in this study. There were significant differences in the appearance of iris vessels among different sex, gestational age and birth weight, postmenstrual age and weight at the time of examination and iris color groups. The iris vessels were more visualized in males relative to females (OR = 6.313, 95% CI 2.529–15.759). The greater the postmenstrual age at the time of examination, the lower the visualization of iris vessels (OR = 0.377, 95% CI 0.247–0.575). In addition, although visualization of anterior chamber angle vessels differed within the birth gestation age and weight at examination groups, there was no significant correlation by regression analysis.

**Conclusions:**

The anterior segment of perinatal neonates can be visualized by the wide-field digital imaging system. The neonatal iris and anterior chamber angle are immature, and the visible vessels at the anterior chamber angle that vanish later than the surface of the iris are characteristic structures.

## Introduction

According to the World Health Organization in 2010, about 1% of children worldwide are affected by vision impairment [1]. Among the causes of visual impairment, congenital cataracts, retinopathy of prematurity, congenital glaucoma, and tumors can preserve vision as much as possible through early detection and treatment. In addition, as medical technology continues to develop, the tremendous advances in perinatal medicine and neonatal emergency medicine have greatly increased the survival rate of many previously difficult-to-treat preterm infants, which further adds to the complexity of neonatal eye disease [2]. Therefore, early and correct diagnosis and timely treatment of neonatal eye diseases can reduce low vision and blindness and reduce the economic burden on society.

There were various methods for neonatal eye examination. The earliest methods used include red reflex testing (RRT) [3], indirect ophthalmoscopy, and ultrasound biometrics. With the development of science and technology, wide-field digital imaging systems [4], handheld optical correlation tomography scanners [5], scanning laser fundus photography systems [6] are also gradually being used in the clinical and scientific research of neonatal eye diseases.

In recent years, the wide-field digital imaging systems (RetCam 3 Natus Medical, Inc., USA) had been used to screen newborns for eye diseases [7, 8]. In particular, retinopathy of prematurity, retinal hemorrhage, familial exudative vitreoretinopathy, abnormal fundus pigmentation, choroidal defect, idiopathic retinal vein tortuosity, exudative changes [9] and other posterior segment diseases. Although the wide-field digital imaging system can focus on the structures of the anterior segment such as the iris and the angle of the chamber by adjusting the focal length, the research in this area is very scarce. This study intends to conduct a cross-sectional study of the ocular anterior segment of neonates by using a wide-field digital imaging system in order to explore the reliability of the examination of anterior segment diseases and to observe the development of anterior segment structures.

## Materials and methods

This is a cross-sectional study that was retrospectively assessed at the Zhongnan Hospital of Wuhan University.

### Ethics statement

Informed consent from the subject’s parents was obtained, and the study was approved by the Ethics Committee of Zhongnan Hospital of Wuhan University, Wuhan, China (Approval No.2,022,115 K). The registration number of the clinical trial center is ChiCTR2200062801 (19/08/2022).

### Data collection

Perinatal neonates within 42 days of delivery undergoing eye examination in the outpatient clinic of Zhongnan Hospital of Wuhan University from April 2022 to July 2022 were collected. All newborns had eye examinations with flashlights, handheld slit-lamps, and wide-angle digital imaging systems. Basic information including sex, gestational age, birth weight, postmenstrual age, and weight at examination as well as previous systemic conditions were also recorded.

Inclusion criteria included: birth weight between 1000 and 4000 g, birth gestational age between 30 and 41 weeks and Han ethnicity, Chinese nationality. Exclusion criteria included: [1] the mother had chromosomal abnormalities and diabetes; [2] the mother had a history of drinking, smoking, and drug use; [3] the newborn had chromosomal abnormalities, severe metabolic disorders and central nervous system infections.

### Widefield digital image system examination

After using mydriatic eye drops (tropicamide 5 mg/mL) to dilate the pupil by about 5 mm, turn on the machine and install a 130° lens. Adjust the focal length and light source (13–38 units) of the lens, disinfect the lens with an alcohol cotton swab, and drop Alcaine (proparacaine hydrochloride 0.5%) into the conjunctival sac for topical anesthesia. Stabilize the subject’s head, open the eyelids with an eyelid opener, and apply carbomer eye gel on the corneal surface. The lens is placed lightly in the center of the cornea, and the angle of the lens is adjusted to collect images of the iris and anterior chamber angle. Considering that the anterior chamber angle shooting is related to the tilt angle of the lens, only the images of the nasal chamber angle with a large operating space are collected. Then the focal length was moved back, and the lens, vitreous and posterior pole, temporal side, nasal side, upper side, and lower side of the retina were taken in turn.

A physician with ten years of clinical experience was responsible for image screening. In addition, the images were considered acceptable only if structure of the iris and anterior chamber angle were clearly distinguishable in follow-up studies.

### Data analysis

Statistical analyses were performed using SPSS 21.0 (IBM Corporation, Armonk, NY, USA). The average gestational age and weight were described as mean ± SD. Student’s t-test was used for continuous variables and χ² test for categorical variables. We also performed logistic regression analyses to determine associations between the characteristic clinical manifestations encountered and other demographic and clinical factors. Variables were selected for the logistic regression models according to a p-value < 0.10 in the univariate analysis. The OR and 95% CI were calculated. A p-value less than 0.05 was considered as statistically significant.

## Results

**Fig. 1:**
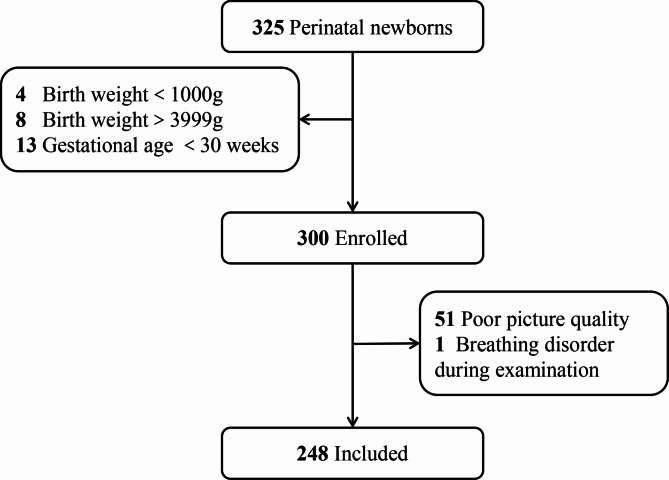
Flow chart of participants included for analysis

During primary screening, 325 perinatal newborns underwent wide-field digital image system examination, of which 248 were included in the study and 77 (23.69%) were excluded (Fig. 1). Of 248 newborns (496 eyes) in the screening study, 48.79% were female. The average gestational age was 35.24 ± 3.04 weeks, and the average birth weight was 2363.11 ± 730.56 g. The postmentrual age and weight at the time of examination were 40.94 ± 3.38 weeks and 3571.86 ± 1164.70 g (Table 1).

**Table 1:** Perinatal characteristics of the study population

Parameter	No. of Patients (n = 248)
Male sex†	127 (51.21)
Female sex†	121 (48.79)
Gestational age at birth (wk)*	35.24 ± 3.04
Birth weight (g)*	2363.11 ± 730.56
Gestational age at examination(wk)*	40.94 ± 3.38
Weight at examination (g)*	3571.859 ± 1164.70
Cesarean section	125 (50.40)

* Data are means ± standard deviation. †Data are No. (%)

Table 2 demonstrates the anterior segment abnormalities detected by ocular examinations of perinatal newborns with the wide-field digital image system. The most common abnormality was visualization of anterior chamber angle vessels (79.03%). Other abnormalities included visualization of iris vessels (51.21%), iris process (42.34%), persistent pupillary membranes (PPM) (19.35%), persistent hyperplasia of primary vitreous (PHPV) (8.47%). We also found albinism, congenital cataracts, corneal leucoma, and subconjunctival hemorrhage with a frequency of 0.81%, 3.23%, 0.4%, and 2.42%.

**Table 2:** Anterior segment abnormalities detected by ocular examination examination of perinatal newborns with the RetCam3

Anterior segment abnormalities	No.(%)
Albinism	2(0.81%)
PHPV	21(8.47%)
PPM	48(19.35%)
Corneal leucoma	1(0.4%)
Congenital cataract	8(3.23%)
Subconjunctival haemorrhage	6(2.42%)
Visualization of iris vessels	127(51.21%)
Visualization of anterior chamber angle vessels	196(79.03%)
Iris process	105(42.34%)

PHPV: Persistent hyperplasia of primary vitreous; PPM: Persistent pupillary membranes;

### Iris structure

**Fig. 2:**
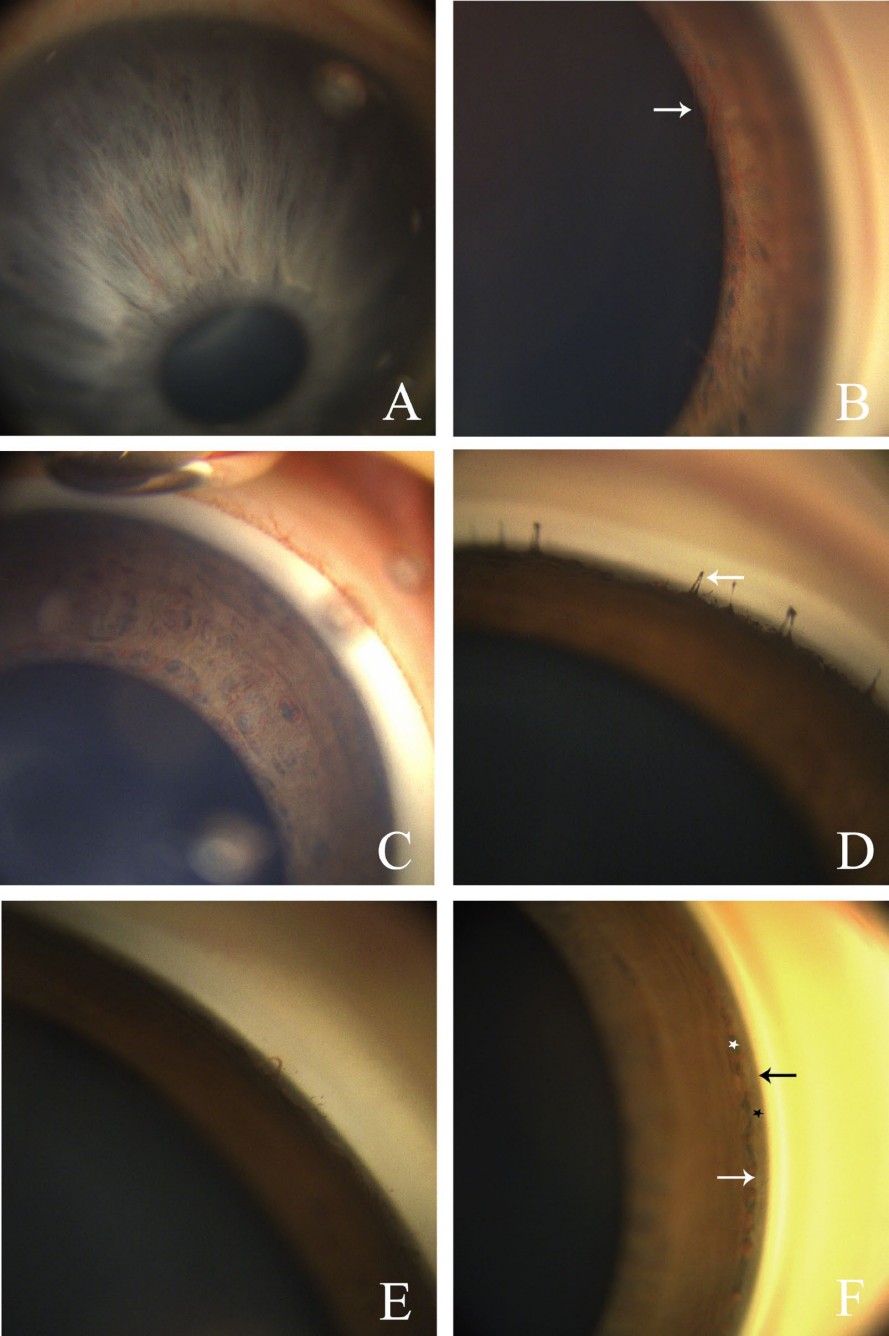
At 42.14w (**A**), the iris with albinism appears light gray. At 36.43w (**B**), the persistent pupillary membranes of the blood vessels(white arrow)were found in the pupil area of the iris. At 36w (**C**), radial blood vessels were seen on the iris surface. At 44w (**D**), iris process was seen at the root of the iris and occasionally isolated blood vessels protruding from the surface of the iris at 39.86 (**E**). At 41w (**F**), the Schwalbe’s line (black arrow) was near the end of the Descemet’s membrane, the trabecular meshwork was dark (black asterisk), and the scleral spur (white arrow) was discontinuous. The iris root blood vessels were clearly apparent, and the dark gray area between the vessels (white asterisks) could be the posterior aspect of the iris pigment membrane, where iris stromal dysplasia or ciliary body band develops. Remarks: All data were at the time of examination gestational age

Three iris colors including brown, light brown and light gray were found in the inspected newborns, of which brown was the most common (70.56%). The iris of newborns with albinism and fairer skin would appear light gray (Fig. 2.A). In the pupillary region of the iris, PPM were frequently observed, the majority of these membranes were filamentous or reticular across the lens and lacked vascular structures, but a few of these membranes could contain vascular structures (Fig. 2.B). Radial blood vessels were seen on the iris surface (Fig. 2.C), and its visualization was statistically significantly different from factors such as gender, iris color, PPM, and PHPV (Table 3). Following logistic regression analysis, the findings revealed that factors such as birth weight at birth and examination, PHPV, and PPM had no impact on the visualization of iris vessels. However, there was a strong correlation between the visualization of iris vessels and gender, gestational age at birth and examination, and iris color (Table 4). The iris vessels were more visualized in males relative to females (OR = 6.313, 95% CI 2.529–15.759). The greater the gestational age at the time of examination, the lower the visualization of iris vessels (OR = 0.377, 95% CI 0.247–0.575). In addition, the lighter the color of the iris, the more obvious the blood vessels in the iris.

**Table 3:** Difference analysis for visualization of iris vessels and anterior chamber angle vessels according to neonatal sociodemographic and clinical characteristics

Characteristics	Total	Iris vessels			Anterior chamber angle vessels		
		No. (%)		*p* value	No. (%)		*p* value
		Positive result	Negative result		Positive result	Negative result	
Sex				0.000**			0.173
Female	121	46(38.02%)	75(61.98%)		100(82.64%)	21(17.36%)	
Male	127	81(63.78%)	46(36.22%)		96(75.59%)	31(24.41%)	
Gestation age at birth (wk)				0.001**			0.041*
30-33.9	94	61(64.89%)	33(35.11%)		73(77.66%)	21(22.34%)	
34-36.9	76	38(50%)	38(50%)		67(88.16%)	9(11.84%)	
37–41	78	28(35.9%)	50(64.1%)		56(71.79%)	22(28.21%)	
Birth weight (g)				0.014**			0.025
1000–1999	98	61(62.24%)	38(38.78%)		82(83.67%)	16(16.33%)	
2000–2999	90	42(46.67%)	48(53.33%)		74(82.22%)	16(17.78%)	
3000–3999	60	24(40%)	36(60%)		40(66.67%)	20(33.33%)	
Gestational age at examination (wk)				0.000**			0.505
34-36.9	42	42(100%)	0(0%)		31(73.81%)	11(26.19%)	
37-40.9	60	35(58.33%)	25(41.67%)		50(83.33%)	10(16.67%)	
41–47	146	50(34.25%)	96(65.75%)		115(78.77%)	31(21.23%)	
Weight at examination (g)				0.000**			0.002**
1400–2999	80	64(80%)	16(20%)		64(80%)	16(20%)	
3000–3999	60	27(45%)	33(55%)		56(93.33%)	4(6.67%)	
4000–6500	108	36(33.33%)	72(66.67%)		76(70.37%)	32(29.63%)	
Iris color				0.000**			0.087
Brown	175	61(34.86%)	114(65.14%)		135(77.14%)	40(22.86%)	
Light brown	56	51(91.07%)	5(8.93%)		44(78.57%)	12(21.43%)	
Light grey	17	15(88.23%)	2(11.76%)		17(100%)	0(0%)	
PHPV	21	19(90.48%)	2(9.52%)	0.000**	15(71.43%)	6(28.57%)	0.371
PPM	48	41(85.42%)	7(14.58%)	0.000**	42(87.5%)	6(12.5%)	0.109

PHPV: Persistent hyperplasia of primary vitreous; PPM: Persistent pupillary membranes; ** p < 0.01;*p < 0.05

**Table 4:** Regression analysis for visualization of iris vessels and anterior chamber angle vessels according to neonatal sociodemographic and clinical characteristics

Characteristics	Iris vessels			Anterior chamber angle vessels		
	*p* value	Raw OR	Raw OR (95% CI)	*p* value	Raw OR	Raw OR (95% CI)
Sex (female)	0.000**	6.313	2.529–15.759	0.13	0.589	0.298–1.168
Gestation age at birth (wk)	0.546	1.142	0.742–1.759	0.245	1.243	0.861–1.795
Birth weight (g)	0.621	1	0.999–1.002	0.081	0.999	0.997 -1
Gestational age at examination (wk)	0.000**	0.377	0.247–0.575	0.847	1.026	0.79–1.332
Weight at examination (g)	0.152	1	1-1.002	0.428	1	0.999-1
Iris color						
Brown	Reference	Reference	Reference	Reference	Reference	Reference
Light brown	0.000**	74.063	18.835-291.232	0.215	1.753	0.721–4.261
Light grey	0.000**	26.751	4.265-167.785	0.999	NA	NA
PHPV	0.895	0.856	0.084–8.707	0.084	4.053	0.828–19.834
PPM	0.632	0.098	0.022–0.429	0.376	0.584	0.178–1.918

PHPV: Persistent hyperplasia of primary vitreous; PPM: Persistent pupillary membranes; ** p < 0.01

### Anterior chamber angle structure

Visualization of angle structures was achieved in all neonates. The most notable feature was the visualization of vascular at the root of the iris. Although the neonatal pupils were all dilated, all angles were open, the trabecular meshwork was fully exposed, and the hyperreflective scleral spur was occasionally visible (Fig. 2.D, E, F). Anterior chamber angle vessels visualization was statistically significantly different from iris and PPM (Table 3), there was no significant correlation by regression analysis. (Table 4).

## Discussion

This study captured the development of the ocular anterior segment in newborns, including the cornea, lens, iris, and anterior chamber angle through the wide-field digital imaging system. The most common anterior segment abnormalities included visualization of anterior chamber angle vessels and iris vessels, iris process, PPM, and PHPV. We regressively analyzed these abnormal structures and neonatal sex, age, weight, etc., summarized the clinical significance of different abnormal anterior segments in the growth process of neonates.

Iris blood supply is composed by the terminal branches of the anterior and long posterior ciliary arteries [10, 11]. The small ring of iris arteries is located 1.5 mm outside the pupillary margin at the pupillary collar, and it is mainly a remnant of embryonic vessels, most of which are occluded. Most iris arteries enter the pupillary margin as capillaries, before folding back to form veins [12]. The iris vasculature is not visible in normal adults with darker pigments. Spierer observed normal White newborns through slit-lamp biomicroscopy and found that iris vascularity correlated significantly with postmenstrual age at examination [13], which was consistent with our study. The visibility of iris vessels in term infants in our research was lower than that of Spierer, which may be due to differences in participants’ races. Differences in iris vessels by sex may be related to inherent physiological differences. Gender differences were reflected in adverse respiratory illness, cardiovascular system, insulin secretion, and infectious diseases from embryonic to infant stages [14]. Choroidal volume, thickness, and circulation [15–17] in adults also show gender differences. The mechanism of this discrepancy requires further study.

The iris vasculature dysfunction has been linked to primary open-angle glaucoma, uveitis and even cataracts [18].In addition, iris vessels of cocaine intoxicated [19] and hypoxic ischemic encephalopathy [20] neonates were more tortuous and dilated. More than half of term infants born to mothers with diabetes have markedly twisted and dilated iris blood vessels in 2 weeks [21]. Our study shows that tortuous iris vessels were more likely to be observed in preterm infants. Preterm birth was associated with iris vessels, suggesting that iris vascularity characterizes the development of preterm infants. In addition, almost all neonates with PPM and PHPV are accompanied by iris vascularity, and this type of embryonic vascular degenerative disease may interact with iris vascular regression in pathogenesis. Iris vasculature which is

 radial and has no obvious branches also needs to be distinguished from neovascularization.

Iris discoloration can be a sign of pathology. Hereditary, biochemical, and infectious lesions can present with iris discoloration [22]. In an experiment of iris color screening of full-term newborns, 80% of Asian newborns had brown irises [22]. We found that, with the exception of a few children with albinism, whose irises were light gray, the neonates in this study were almost all brown, and the irises of preterm infants were even lighter. Although changes in melanin production can lead to changes in iris color during infancy over time [23], it seems unlikely that brown irises will change much [24]. PPM are thought to be derived from the tunica vasculosa lentis (TVL) and have normally disappeared by the 34th week of gestation [25]. This anterior segment abnormality may be associated with possible intrauterine infection [26]. Our incidence of PPM in perinatal neonates was lower than 95% in the Tanzer [27] study, indicating rapid degeneration of perinatal PPM. In addition, PPM, which significantly affects visual pathways, was not significantly correlated with gestational age at birth, and eye screening should be recommended for all newborns including term infants.

When the anterior chamber angle develops normally during fetal stage, the ciliary body attachment from Schwalbe’s line (20 weeks) to the scleral spur (36 weeks), and then to a location behind the scleral spur (postnatally) [28, 29]. According to the histological study of Douglas [30], the anterior chamber angle of preterm infants in this study was open, the scleral spur was visible partially (34 weeks), and it turned more widely exposed at 47 weeks. We thought that although the neonatal anterior angle was structurally immature, the high-gloss white scleral spur, which was easily observed in front of the ciliary body in wide-field digital imaging system anterior angle photography, was important as an anatomical marker to assess the opening of the neonatal anterior chamber angle. The amount of pigmentation of the trabecular meshwork is important in the diagnosis of different glaucoma [31]. On anterior chamber angle examination in children older than 5 years by wide-field digital imaging system, the trabecular mesh was clearly pigmented [32]. However, because of the tiny angle between the irid-cornea, the trabecular meshwork was all shown dark in the image, which cannot clearly distinguish more details by increasing the brightness, according to our observation. Similar to the mammalian pectinate ligament, the iris process exists in the form of iris stroma or collagen covered by variably pigmented mesothelium stretching from the iris root to peripheral Descemet’s membrane [33]. The iris process can be filamentous, rod-shaped, or reticular, and it is rare in healthy adults. Ellis suggested that infantile glaucoma resulted from a congenital remnant of the pectinate ligament [34]. Severing the pectinate ligament can increase aqueous humor outflow in congenitally glaucomatous eyes [35, 36]. Previous studies have suggested that the iris process may be associated with pigmented glaucoma and developmental glaucoma. However, the present study found that the iris process was very common in perinatal neonates, and these neonates also had normal intraocular pressure and optic disc. Therefore, no conclusion can be drawn that the iris process is necessarily related to developmental glaucoma.

Vessels were visible at the iris root in almost all anterior chamber angles, inserting radially into the vascular annulus deep to the iris. Since anterior chamber angle vessels visualization was so common, we might consider it typical of neonatal anterior chamber angle. The visualization of iris vessels on the surface of the iris were significantly connected to gestational age at the time of examination. However, the visualization of anterior chamber angle vessels had no relationship with gestational age at the time of examination. It is possible that the iris root matrix and blood vessels develop much later than the iris surface matrix and blood vessels (more than 47 weeks). The dark gray area between the vessels at the anterior chamber angles may be the posterior surface of the iris pigment membrane where iris stromal dysplasia manifests or the ciliary body band. The width of the ciliary body band indicator of the development of the anterior chamber angle. An invisible or very narrow ciliary body band represents an underdevelopment of the iridocorneal angle [37, 38]. The ciliary body band of all perinatal neonatals in our study were underdeveloped.

Our study was subjected to several limitations that need to be addressed. The precision of our single-institution al

study was limited because it had a moderate sample size. Our study population was racially restricted, so the results may not be generalizable to more diverse groups. Mothers and infants with severe disease were excluded from the included investigators, and the incidence of anterior segment abnormalities may have been biased. In addition, the correlation between fundus disease and anterior segment disease was not analyzed in the studies, so we also need a larger sample and more detailed analysis to help with clinical diagnosis and treatment. Lastly, this technique requires more training and is more operator dependent. We intended our results to be generalizable to the general practice.

In conclusion, the anterior segment structure of the perinatal newborn has its own characteristics and is clearly different from that of children. The fast and non-invasive wide-field digital imaging system allows us to observe and record these features. It fills the gap of direct anterior segment angle examination of newborns, and future studies of anterior segment angle development based on this technology will provide a strong basis for the pathogenesis of glaucoma.

## Data Availability

Corresponding author will provide data when editors, reviewers or readers need it.
